# Recent Advances in Functional Biopolymer Films with Antimicrobial and Antioxidant Properties for Enhanced Food Packaging

**DOI:** 10.3390/polym17091257

**Published:** 2025-05-05

**Authors:** Thirukumaran Periyasamy, Shakila Parveen Asrafali, Jaewoong Lee

**Affiliations:** Department of Fiber System Engineering, Yeungnam University, Gyeongsan 38541, Republic of Korea; thirukumaran@ynu.ac.kr (T.P.); shakilaparveen@yu.ac.kr (S.P.A.)

**Keywords:** cellulose, bio-active fillers, functional biopolymers, sustainable food packaging

## Abstract

Food packaging plays a crucial role in preserving freshness and prolonging shelf life worldwide. However, traditional packaging primarily acts as a passive barrier, providing limited protection against spoilage. Packaged food often deteriorates due to oxidation and microbial growth, reducing its quality over time. Moreover, the majority of commercial packaging relies on petroleum-derived polymers, which add to environmental pollution since they are not biodegradable. Growing concerns over sustainability have driven research into eco-friendly alternatives, particularly natural-based active packaging solutions. Among the various biopolymers, cellulose is the most abundant natural polysaccharide and has gained attention for its biodegradability, non-toxicity, and compatibility with biological systems. These qualities make it a strong candidate for developing sustainable packaging materials. However, pure cellulose films have limitations, as they lack antimicrobial and antioxidant properties, reducing their ability to actively preserve food. To tackle this issue, researchers have created cellulose-based active packaging films by integrating bioactive agents with antimicrobial and antioxidant properties. Recent innovations emphasize improving these films through the incorporation of natural extracts, polyphenols, nanoparticles, and microparticles. These enhancements strengthen their protective functions, leading to more effective food preservation. The films are generally classified into two types: (i) blend films, where soluble antimicrobial and antioxidant substances like plant extracts and polyphenols are incorporated into the cellulose solution, and (ii) composite films, which embed nano- or micro-sized bioactive fillers within the cellulose structure. The addition of these functional components enhances the antimicrobial and antioxidant efficiency of the films while also affecting properties like water resistance, vapor permeability, and mechanical strength. The continuous progress in cellulose-based active packaging highlights its potential as a viable alternative to conventional materials. These innovative films not only extend food shelf life but also contribute to environmental sustainability by reducing reliance on synthetic polymers. This review deals with the development of functional biopolymer films with antimicrobial and antioxidant properties towards sustainable food packaging.

## 1. Introduction

Food spoilage remains a significant challenge, with approximately 30% of food wasted during harvest and postharvest transportation. One of the most effective ways to mitigate this issue is through the use of advanced food packaging solutions. Packaging serves as a protective barrier against external factors such as microbial contamination, mechanical damage, and ultraviolet (UV) radiation, ensuring food safety and extending shelf life. Traditionally, petroleum-based materials have dominated the food packaging industry due to their superior mechanical properties, chemical stability, and effective barrier functionalities. However, these materials are derived from nonrenewable resources, contribute to environmental pollution, and pose significant ecological and economic challenges due to their nonbiodegradability [1,2,3,4,5]. As sustainability and environmental concerns gain momentum, researchers have been actively developing biodegradable, renewable, and eco-friendly food packaging alternatives. Biopolymer-based films, derived from natural sources such as polysaccharides, lipids, and proteins, offer a promising alternative to conventional plastics. However, these materials frequently exhibit weaknesses in mechanical durability and barrier effectiveness. To overcome these challenges, biopolymer-based food packaging has increasingly incorporated antimicrobial and antioxidant agents to enhance its functionality. Such functional biopolymer films not only provide protection against spoilage but also enhance the shelf life of packaged food products. Biopolymers such as polysaccharides, proteins, and lipids have been widely studied for their potential in sustainable food packaging solutions. Among these, polysaccharides such as cellulose, starch, chitosan, and carrageenan have shown remarkable biodegradability and renewability. Lipid-based materials like waxes and fatty acids, along with protein-based materials such as casein and soy protein, have also demonstrated considerable promise in food packaging applications. Although biopolymer-based films offer advantages, their mechanical and barrier properties are typically weaker than those of petroleum-based plastics. These limitations have led to the development of composite films reinforced with active components to enhance their functional properties [6,7,8,9].

To improve the efficacy of biopolymer-based films, functional additives are incorporated to impart antimicrobial and antioxidant properties. These additives can be categorized into two primary groups: synthetic and natural bioactive compounds. While synthetic additives effectively regulate the packaging atmosphere, inhibit microbial growth, and control moisture content, their safety must be rigorously assessed due to potential health risks. Natural bioactive compounds, including plant-derived polyphenols, essential oils, and antimicrobial peptides, are increasingly preferred for their biocompatibility and eco-friendly nature. Antioxidant materials such as nanofillers and polyphenols play a crucial role in preventing oxidation, thereby extending food shelf life. Similarly, antimicrobial agents such as silver nanoparticles, chitosan derivatives, and natural plant extracts have been incorporated into packaging films to combat microbial contamination. The integration of such bioactive compounds enhances the protective capabilities of biopolymer films, making them viable alternatives to synthetic food packaging materials [10,11,12,13,14].

Improved food packaging stands out not only as a barrier to external contamination but also as a multifunctional strategy that interacts with other preservation techniques. The unique advantages of advanced food packaging, especially when compared to other methods like irradiation, preservatives, or cold chain logistics, are given in Table 1. Advanced fabrication methods have been utilized to improve the performance of biopolymer-based food packaging. One of the widely studied approaches is the incorporation of cellulose and its derivatives in active packaging systems. Cellulose is the most abundant natural polymer on Earth and possesses excellent film-forming capabilities. Various cellulose derivatives, such as cellulose nanocrystals and bacterial cellulose, have been explored for food packaging applications. These materials can be modified chemically or physically to enhance their barrier properties and mechanical strength. Chitosan, another prominent biopolymer, is derived from chitin found in crustaceans, fungi, and insects. It is well known for its biocompatibility, biodegradability, and intrinsic antimicrobial properties. However, chitosan has limited solubility in neutral or alkaline conditions, necessitating chemical modifications to improve its applicability in food packaging. Modified chitosan derivatives, including quaternary ammonium chitosan and carboxymethyl chitosan, have demonstrated enhanced antimicrobial efficacy and mechanical strength. Additionally, chitosan-based composites combined with nanoparticles, essential oils, and plant extracts have shown promising results in developing active packaging solutions [15,16,17,18,19,20,21]. While biopolymer-based food packaging offers significant advantages in sustainability and functionality, certain concerns must be addressed. The potential migration of nanoparticles and bioactive compounds into food raises safety considerations. For instance, the release of silver nanoparticles from antimicrobial packaging films could pose toxicity risks. Similarly, the leaching of certain synthetic additives may have adverse health effects. To mitigate these risks, regulatory agencies require thorough assessments of new food packaging materials to ensure compliance with safety standards. Additionally, large-scale production of biopolymer films must be optimized for economic feasibility. The high cost of raw materials and processing techniques currently limits their widespread adoption. Biopolymers are costlier than petroleum-based materials due to lower production volumes, costly extraction and purification methods, and seasonal and regional availability. For example, LDPE costs about USD 1.5–2.0 per kg, whereas PLA costs about USD 2.5–5.0 per kg and high-purity chitosan costs about USD 15–30 per kg. Research efforts are focused on developing cost-effective fabrication methods and exploring abundant natural resources for sustainable packaging solutions. The development of next-generation food packaging materials is driven by the increasing demand for sustainable, biodegradable, and functional solutions. Future research should focus on improving the mechanical properties and stability of biopolymer films while ensuring their safety for food contact applications. Additionally, advancements in nanotechnology, bioengineering, and material science can further enhance the functionality of biopolymer-based packaging [16,19,21]. This review aims to serve both academic researchers and industry professionals by providing a comprehensive yet critical synthesis of recent developments in the field rather than merely compiling the existing literature. Several sections have been included to better integrate and analyze prior studies, identifying trends, gaps, and opportunities for future work and application.

A key challenge remains in balancing cost-effectiveness with performance. The sustainable sourcing of raw materials, efficient manufacturing processes, and regulatory compliance are crucial factors that will determine the commercial viability of biopolymer-based food packaging. Moreover, consumer acceptance and market readiness will play a significant role in driving the adoption of these materials on a larger scale. The shift towards sustainable food packaging has paved the way for the development of functional biopolymer films with antimicrobial and antioxidant properties. Biopolymer-based films derived from polysaccharides, proteins, and lipids have demonstrated significant potential as eco-friendly alternatives to traditional plastic packaging. The incorporation of bioactive compounds further enhances their protective capabilities, extending food shelf life and ensuring safety. However, challenges such as mechanical limitations, production costs, and regulatory considerations must be addressed to enable widespread implementation. Continued research and innovation in biopolymer film fabrication will play a pivotal role in the future of sustainable food packaging [30,31,32,33,34].

### 1.1. Classification of Films: Biodegradable Films

Biodegradable films are an eco-friendly alternative to conventional plastics, designed to break down naturally into non-toxic byproducts such as carbon dioxide, methane, and water. These films are typically made from natural polymers, including polysaccharides like starch, cellulose, and chitosan, as well as proteins such as gelatin, casein, and soy protein. Lipids like waxes and fatty acids are often incorporated to improve water resistance. While these films significantly reduce environmental pollution, they often have weaker mechanical properties and higher water permeability compared to synthetic plastics. To improve flexibility, plasticizers like glycerol and sorbitol are added, though they may also increase moisture sensitivity. Despite these challenges, biodegradable films remain a crucial part of sustainable packaging solutions [5,13,27].

### 1.2. Edible Films and Coatings

Edible films and coatings, a subset of biodegradable films, are designed to be safely consumed along with the packaged food. These films are typically made from food-grade biopolymers such as starch, pectin, alginate, and gelatin. Lipids and essential oils are often incorporated to improve their water resistance and antimicrobial properties. Unlike conventional packaging, edible films provide a direct protective layer over the food, reducing the need for additional packaging materials. They are particularly effective for fresh produce, dairy, and meat products, preventing moisture loss, oxidation, and microbial contamination. Their application not only extends shelf life but also contributes to reducing plastic waste in the food industry [35,36,37].

### 1.3. Active Packaging Films

Active packaging films interact directly with food to enhance safety and extend shelf life by incorporating bioactive compounds. These films function either by releasing beneficial substances such as antimicrobial agents and antioxidants or by absorbing harmful compounds like oxygen, moisture, and ethylene gas. Chitosan, polylactic acid (PLA), and other nanocomposite biopolymers are commonly used in active packaging due to their compatibility with bioactive components. Essential oils, polyphenols, and metal nanoparticles such as silver and zinc oxide are often added to enhance antimicrobial properties. Active packaging is especially beneficial for perishable foods, as it prevents oxidation and microbial spoilage, ensuring longer-lasting freshness [38,39,40,41,42,43,44].

### 1.4. Intelligent Packaging Films

Intelligent packaging films incorporate sensors or indicators that monitor food quality in real time. These films react to environmental changes such as pH fluctuations, oxygen exposure, temperature variations, and microbial growth, providing visual or electronic signals about food safety. Common intelligent packaging technologies include time-temperature indicators (TTIs) that track thermal exposure, food freshness indicators (FFIs) that detect spoilage-related gases, and pH-sensitive films that change color based on acidity levels. Many pH-sensitive films use natural pigments such as anthocyanins from red cabbage, which shift in color as food deteriorates. Advances in biosensor technology have further improved intelligent films, making them highly effective in monitoring perishable goods such as seafood, dairy, and meat products [45,46,47,48,49,50,51].

### 1.5. Multilayer and Composite Films

Multilayer and composite films are designed to enhance the mechanical strength, barrier properties, and overall functionality of biopolymer-based packaging. Composite films combine different biopolymers, such as proteins and polysaccharides, to improve flexibility, durability, and moisture resistance. Multilayer films, on the other hand, stack different materials to create controlled permeability and superior protection against external factors like humidity and oxygen. These advanced film structures allow for the simultaneous incorporation of antimicrobial and antioxidant agents, making them highly effective in food preservation. By integrating nanoparticles, essential oils, or lipid coatings, multilayer and composite films overcome the limitations of single-layer biopolymer films, including lower mechanical strength, poor moisture and gas barrier properties, reduced shelf life, high production costs, and limited compatibility with certain food types, making them a promising solution for high-performance food packaging [52,53,54,55,56].

## 2. Advances in Biopolymer Films for Antimicrobial and Antioxidant Food Packaging

### 2.1. Advanced Fabrication Methods for Enhancing Biopolymer-Based Food Packaging

The development of biodegradable, intelligent packaging materials has become a crucial research focus as an alternative to petroleum-based plastics. Wang et al. (2024) [57] presented a novel approach for fabricating regenerated cellulose/curcumin composite films with Janus structures, utilizing a pH-driven dispersion method and unilateral hydrophobic modification. The study aimed to overcome the limitations of traditional biopolymer-based packaging, such as poor mechanical strength, high moisture sensitivity, and limited gas barrier properties.

#### 2.1.1. Fabrication of Cellulose/Curcumin Composite Films

**Figure 1:**
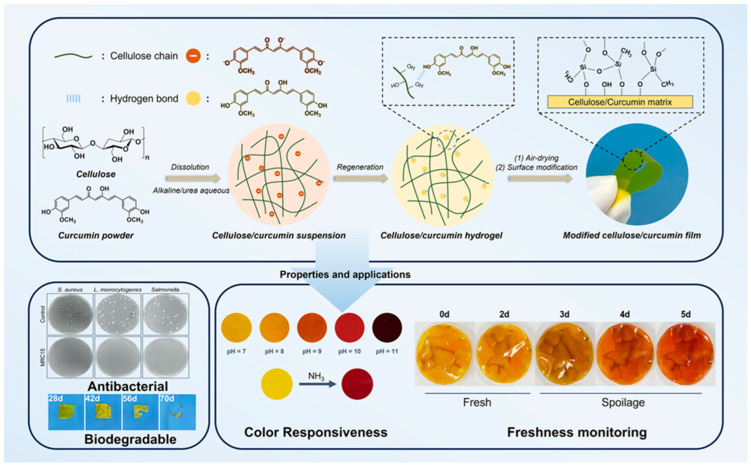
Schematic representation of the modified cellulose/curcumin film (MRC) fabrication through a two-step process: curcumin incorporation into the cellulose matrix, followed by organosilane-based hydrophobic modification. Mechanical flexibility of MRC15, including bending, folding, twisting, and knotting. Performance comparison of PE, PBAT, PLA, and MRC15. SEM images of RC0, RC15, MRC0, and MRC15 [57].

The researchers employed an aqueous alkali/urea solvent system to dissolve cellulose and curcumin, leveraging the pH-sensitive properties of curcumin to achieve uniform dispersion. Unlike conventional methods that require complex solvent systems or high temperatures, this approach allowed for a homogeneous composite film with enhanced stability (Figure 1). Fourier-transform infrared spectroscopy (FTIR) confirmed the successful incorporation of curcumin into the cellulose matrix, with characteristic absorption peaks indicating strong hydrogen bonding interactions between the two components. To further enhance the water resistance of the films, a unilateral hydrophobic modification was performed using the chemical vapor deposition (CVD) of methyltrichlorosilane (MTCS). This resulted in a Janus structure, where one side of the film exhibited hydrophobic properties while the other retained its hydrophilic nature. The scanning electron microscopy (SEM) images revealed that the coated surface formed a rough, hydrophobic layer, resembling a “fish-scale” pattern, while the uncoated side maintained a smooth texture. X-ray photoelectron spectroscopy (XPS) and energy-dispersive X-ray spectroscopy (EDX) confirmed the uniform attachment of organo-silane molecules on the film’s surface, improving its moisture resistance without compromising mechanical properties.

#### 2.1.2. Physical and Functional Properties of the Films

The fabricated films demonstrated excellent optical transparency, with over 80% transmittance in the visible light spectrum (400–800 nm). However, the incorporation of curcumin significantly enhanced UV-blocking properties, reducing transmittance at 280 nm from 80.05% (for pure cellulose films) to just 1.85% (for curcumin-loaded films). This improvement is attributed to the strong UV absorption of curcumin, which provides additional protection against light-induced food degradation. Thermal stability analysis, performed using thermogravimetric analysis (TGA), indicated that the composite films exhibited higher decomposition temperatures than pure cellulose films. The addition of curcumin contributed to an increase in maximum thermal degradation temperature from 297.8 °C (MRC0) to 302.8 °C (MRC10), suggesting improved heat resistance due to enhanced intermolecular interactions. Mechanical testing revealed that the tensile strength of the films improved significantly with curcumin incorporation. The highest strength was observed for MRC15, which exhibited a tensile strength of 73 MPa and an elongation at break of 28.5%, outperforming many conventional biopolymer-based packaging materials. This enhancement is likely due to the reinforcing effect of curcumin, which acts as a natural plasticizer, improving film flexibility while maintaining high tensile strength. Water vapor permeability (WVP) and gas permeability tests confirmed that the Janus films exhibited improved barrier properties. The oxygen permeability of the curcumin-modified films decreased from 3.83 cm^3^/m^2^·24 h·0.1 MPa (RC0) to 2.97 cm^3^/m^2^·24 h·0.1 MPa (MRC15), indicating reduced oxygen diffusion and extended food preservation potential. Similarly, the water contact angle increased significantly after hydrophobic modification, reaching 110.47° for MRC15, demonstrating strong water repellency [57,58].

#### 2.1.3. Intelligent pH-Responsive Freshness Monitoring

**Figure 2:**
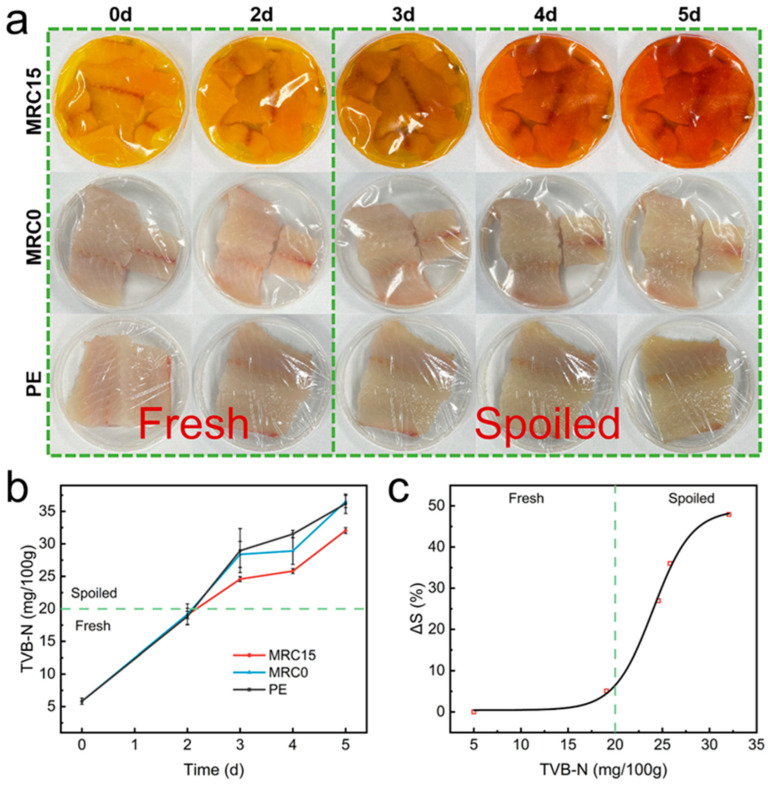
Film color variations in response to fish filets over time (**a**). TVB-N value fluctuations during fish filet storage (**b**). Relationship between TVB-N levels and color shift (ΔS) (**c**) [57].

A key feature of the composite films was their ability to function as intelligent freshness indicators based on pH changes. The researchers observed that curcumin, when incorporated into the films, maintained its pH-sensitive properties. UV–Vis spectroscopy confirmed a redshift in the absorption peak from 425 nm to 465 nm as the pH increased, correlating with a visible color change from bright yellow (pH 7) to reddish brown (pH 11). To test the films’ ability to monitor food freshness, the researchers conducted a fish spoilage study using tilapia filets. The films were used as packaging materials, and their color was monitored over five days at 6 °C. The results showed a gradual color shift from yellow to reddish brown, corresponding to increasing levels of total volatile basic nitrogen (TVB-N)—a key indicator of fish spoilage (Figure 2a,b). By the fifth day, the TVB-N levels in fish packaged with MRC15 were significantly lower than those in commercial polyethylene (PE) packaging, demonstrating the film’s dual role in freshness monitoring and spoilage prevention. Additionally, the composite films exhibited sensitivity to ammonia vapors, with a rapid color change observed within 8 min of exposure to a 0.8 M ammonia solution. The response sensitivity (ΔS) reached 76.6% within this timeframe, indicating potential applications in real-time food quality assessment [57,58].

#### 2.1.4. Antimicrobial and Biodegradability Performance

The study further assessed the antimicrobial properties of the films against three common foodborne pathogens: *Salmonella, Staphylococcus aureus*, and *Listeria monocytogenes*. The antibacterial activity, measured using the ISO-22196 standard, showed a 3.16 log CFU/cm^2^ reduction for *Salmonella*, indicating strong antimicrobial effects. This is attributed to curcumin’s natural antibacterial properties, which disrupt bacterial cell membranes and inhibit microbial growth. Biodegradability tests demonstrated that the composite films completely degraded within 98 days under natural soil conditions (25–35 °C, 50–80% relative humidity). SEM images of the films over time revealed progressive surface erosion, confirming microbial degradation. This highlights the environmental sustainability of the fabricated films, making them a viable alternative to traditional plastic-based food packaging.

### 2.2. In Situ Fruit Packaging with Carboxymethyl Chitosan and Polycaprolactone

Shen et al. (2024) [59] developed an innovative in situ fruit packaging system using solution blow spinning (SBS) technology to improve the preservation of fresh produce. The study addressed key challenges in nanofiber-based packaging, particularly its low production efficiency and weak adhesion to fruit surfaces. Carboxymethyl chitosan (CMCH) and polycaprolactone (PCL) were selected as the primary biopolymer materials due to their biocompatibility, biodegradability, and mechanical flexibility. The study demonstrated a successful application of this method on cherry tomatoes, a fruit highly susceptible to fungal contamination. The in situ packaging method enabled the direct deposition of CMCH/PCL nanofibers onto fruit surfaces within eight minutes, forming a stable yet easily peelable protective coating that significantly reduced fruit deterioration and extended shelf life.

#### 2.2.1. Fabrication and Characterization of In Situ Packaging

The SBS method used in this study offers a significant advantage over electrospinning, which is widely used for nanofiber production but suffers from low efficiency and the need for high-voltage equipment. Unlike electrospinning, SBS does not require an electric field, allowing for high-speed fiber deposition and making it a safer and more scalable alternative for industrial applications. The study incorporated bioactive agents into the nanofiber matrix, including curcumin (CUR), natamycin (NAT), nisin (NIS), and thymol (THY), all of which exhibit antimicrobial properties. The SEM images revealed that the nanofibers had uniform diameters ranging from 242.3 to 327.8 nm, with a well-distributed porous structure. FTIR analysis confirmed the successful encapsulation of active compounds without compromising the integrity of the nanofiber matrix. Additionally, X-ray diffraction (XRD) demonstrated a semi-crystalline structure, and thermogravimetric analysis (TGA) indicated improved thermal stability upon the incorporation of natamycin and curcumin. Mechanical testing revealed that the tensile strength and elasticity of the nanofiber films increased significantly with curcumin and natamycin, making them more durable for food packaging applications [59,60].

**Figure 3:**
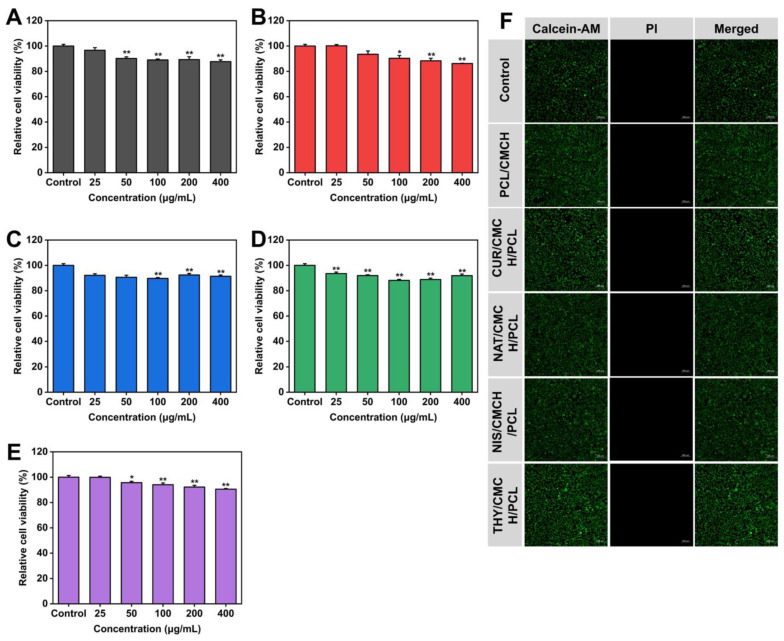
Relative viability of L929 cells after 24 h co-incubation with nanofibrous films: (**A**) CMCH/PCL, (**B**) CUR/CMCH/PCL, (**C**) NAT/CMCH/PCL, (**D**) NIS/CMCH/PCL, and (**E**) THY/CMCH/PCL. (**F**) Fluorescence imaging of cells, where live cells (Calcium-AM) emit green fluorescence, and dead cells (PI) appear red [59]. * and ** indicate statistical significance.

To evaluate the water resistance of the nanofibers, the researchers conducted water contact angle (WCA) measurements. Hydrophobicity is a critical factor in food packaging as it prevents moisture penetration and microbial attachment. The thymol-infused films exhibited the highest hydrophobicity with a WCA of 135.4°, while the other films also showed significant resistance to moisture absorption. Cell cytotoxicity assays (MTT tests) confirmed (Figure 3A–F) that all nanofiber films maintained over 86% cell viability, indicating their biocompatibility and safety for food applications.

#### 2.2.2. Effectiveness of In Situ Packaging on Fruit Preservation

The effectiveness of the in situ packaging system was tested on cherry tomatoes inoculated with *Botrytis cinerea*, a major postharvest fungal pathogen. The results demonstrated a substantial reduction in fungal infection. The untreated tomatoes developed an average lesion diameter of 14.7 mm after 14 days, whereas tomatoes coated with the in situ packaging containing natamycin exhibited an average lesion diameter of just 3.7 mm, highlighting its strong antifungal properties. Beyond fungal inhibition, the in situ packaging significantly reduced weight loss by limiting moisture loss and respiration. The coated tomatoes exhibited lower weight loss throughout a 24-day storage period, while also maintaining firmness, a key quality attribute that influences consumer acceptance. Firmness degradation is typically associated with the enzymatic breakdown of cell wall components during ripening, and the packaging appeared to slow this process. Additionally, color analysis showed that the coated tomatoes experienced delayed pigmentation changes, with lower a* values and higher hue angles compared to the control group, indicating a slower ripening process.

**Figure 4:**
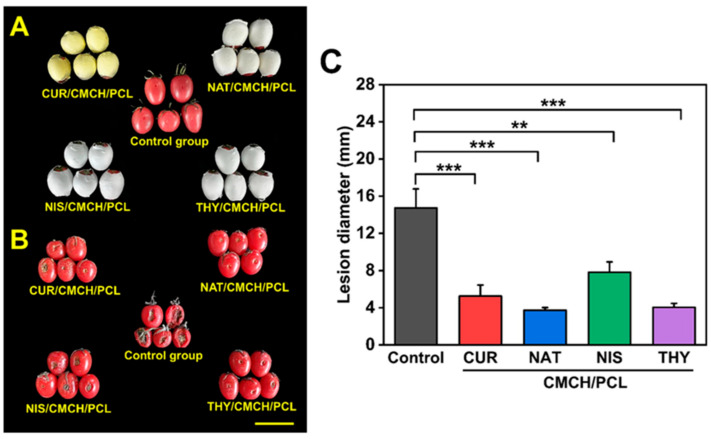
(**A**) Cherry tomatoes infected with Botrytis cinerea and covered with an in situ packaging coating. (**B**) Cherry tomatoes after the removal of the coating. (**C**) Measurement of lesion diameters. Scale bar: 3 cm [59]. ** and *** indicate statistical significance.

To further understand the biochemical changes induced by the in situ packaging, the researchers performed a metabolomic analysis using ultra-performance liquid chromatography and tandem mass spectrometry (UPLC-MS/MS). A total of 1870 metabolites were identified, spanning flavonoids, phenolic acids, alkaloids, lipids, and amino acids. Principal component analysis (PCA) revealed distinct metabolic differences between coated and uncoated tomatoes. The KEGG pathway enrichment analysis indicated that flavonoid biosynthesis was significantly downregulated in coated tomatoes (Figure 4A–C), suggesting that the in situ packaging suppressed oxidative stress-related metabolic processes. Additionally, higher levels of organic acids were detected in packaged tomatoes, further supporting the conclusion that the packaging system slowed metabolic degradation and prolonged fruit freshness [59,60,61].

#### 2.2.3. Industrial Applications and Future Perspectives

Compared to conventional packaging materials, which act as passive barriers, the in situ packaging system developed in this study serves as an active preservation system, incorporating antimicrobial and antioxidant functionalities. Unlike traditional electrospun nanofiber films, which have struggled with low production efficiency and detachment issues, the SBS-based in situ packaging system demonstrated strong adhesion, improved material utilization, and faster processing times, making it highly suitable for industrial-scale applications. Cost analysis estimated that the production cost of in situ packaging per cherry tomato ranged from CNY 0.34 to CNY 0.67 (USD 0.046–0.092), making it a cost-effective alternative, particularly for high-value fruit preservation. The study suggested that large-scale production could further reduce costs, making this approach more competitive with traditional plastic-based packaging materials.

Shen et al. successfully developed a rapid and effective in situ packaging system using SBS technology, demonstrating its potential in fruit preservation. The CMCH/PCL-based nanofibers, functionalized with antimicrobial and antioxidant agents, exhibited enhanced mechanical properties, improved hydrophobicity, and strong biodegradability. The in situ packaging system not only reduced fungal infection and weight loss but also significantly slowed fruit ripening by modulating key metabolic pathways. This study provides a scalable and sustainable alternative to conventional food packaging materials, with potential applications across various fresh produce industries. Future research should focus on optimizing large-scale production methods, extending the applicability to different fruit types, and assessing the long-term environmental impact of the packaging system.

The application of bio-based and biodegradable packaging systems integrated with antimicrobial and antioxidant agents is gaining momentum in industrial settings, driven by increasing consumer demand for natural, sustainable, and safe food preservation solutions. Several companies and research-industry collaborations have begun incorporating bioactive compounds such as essential oils (e.g., thymol, eugenol), metal nanoparticles (e.g., silver, zinc oxide), and plant-derived polyphenols into biodegradable polymer matrices to develop active packaging films. For example, antimicrobial packaging films containing natural extracts from oregano or clove have been used in meat and poultry packaging trials to reduce microbial growth and extend shelf life. Tetra Pak and Amcor have explored bio-based materials embedded with antioxidants to protect lipid-rich foods from oxidation. Likewise, packaging prototypes utilizing chitosan-based films infused with green tea extract or grapefruit seed extract have been piloted for fruits, cheese, and seafood applications. Despite these promising developments, the widespread industrial adoption of such packaging solutions is still limited due to several challenges. Regulatory approvals for food contact materials containing active agents vary significantly by region and are often time consuming and costly. Additionally, the scalability of production, cost-effectiveness, material performance (e.g., barrier properties, mechanical strength), and consumer acceptance remain key barriers to commercialization. Nevertheless, the field continues to evolve rapidly with increasing investment in R&D. Programs like the EU Horizon 2020 and USDA-funded initiatives are actively supporting the development of bioactive packaging technologies. Future trends are likely to include the integration of smart packaging elements (e.g., sensors or freshness indicators), multi-functional films, and biodegradable composites tailored for specific food categories. To ensure practical relevance, it is essential that future research continues to align with industrial requirements, including compatibility with existing packaging lines and recyclability or compostability under real-world conditions.

### 2.3. Development of Eco-Friendly Polylactic Acid/Thermoplastic Starch Films Enhanced with Clove Essential Oil and Cochineal for Active and Intelligent Food Packaging

**Figure 5:**
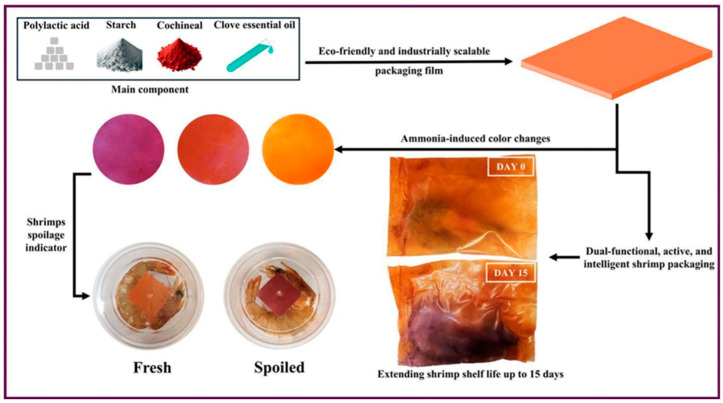
Schematic illustration of the film fabrication process [62].

Mohammadi and Fasihi (2025) [62] developed an eco-friendly, dual-functional food packaging film based on polylactic acid (PLA) and thermoplastic starch (TPS), incorporating clove essential oil (CEO) and cochineal for both active and intelligent food preservation. This study aimed to address the limitations of conventional biodegradable packaging by integrating pH-sensitive spoilage indicators and antimicrobial properties, enhancing both food safety and shelf life. The PLA/TPS films were engineered to respond to volatile nitrogenous compounds (ammonia, trimethylamine) released during seafood spoilage, with real-time color changes indicating freshness. Modified TPS with citric acid improved film flexibility, interfacial adhesion, and mechanical properties, making the material suitable for industrial-scale food packaging (Figure 5). The films were tested for ammonia sensitivity, antibacterial activity, mechanical performance, and effectiveness in extending shrimp shelf life under refrigeration.

#### 2.3.1. Fabrication and Structural Characterization of the PLA/TPS Films

The PLA/TPS-based packaging films were produced using melt blending and hot pressing, ensuring compatibility with standard industrial film manufacturing techniques. Unmodified TPS presented poor interfacial adhesion due to its hydrophilic nature, leading to phase separation when blended with PLA. To improve compatibility, citric acid-modified TPS (MTPS) was used, enhancing hydrogen bonding interactions and enabling better dispersion within the PLA matrix. FTIR confirmed successful esterification between citric acid and starch, which improved mechanical strength and flexibility. SEM images revealed that PLA/TPS blends with modified starch exhibited fewer voids, better phase dispersion, and improved structural integrity, while XRD analysis showed increased crystallinity, contributing to improved moisture barrier properties [62,63].

Colorimetric analysis demonstrated that cochineal, a natural anthraquinone dye, provided pH-responsive color shifts, essential for real-time freshness monitoring in food packaging applications. Cochineal exhibited an orange-to-purple transition in response to ammonia exposure, with a 117% greater color change (ΔE = 69.54) compared to unmodified PLA/TPS films. Films containing 20% modified TPS (PM20C) achieved faster and more uniform color changes within 60 min, significantly outperforming traditional ammonia-sensitive polymer films.

#### 2.3.2. Antibacterial Activity and Food Preservation Performance

**Figure 6:**
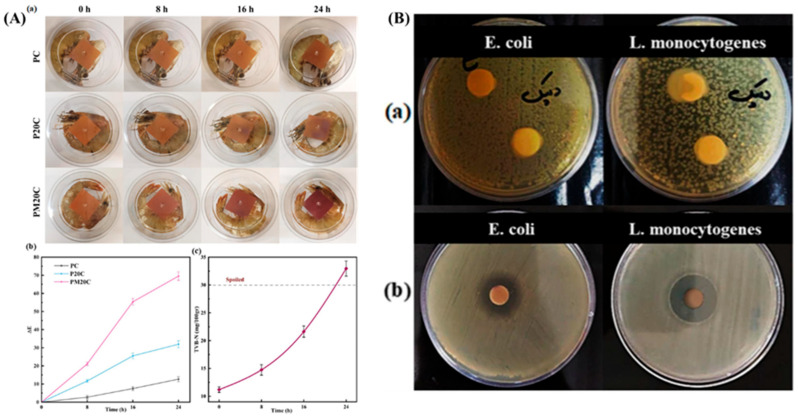
(**A**) (a) Indicator films used for shrimp freshness monitoring, (b) color variation analysis, and (c) TVBN value changes over different storage durations. Scale bar: 3 cm. (**B**) Antibacterial activity of films against *E. coli* and *L. monocytogenes*: P (a) and PM20C (b) [P = PLA:TPS (100:0) PLA (polylactic acid) and TPS (thermoplastic starch), PC = PLA:TPS (100:0) with the addition of cochineal (0.5 Phr), P20C = PLA:TPS (80:20) with the addition of cochineal (0.5 Phr) and PM20C = same composition as P20C, ‘M’ indicates the premodification of TPS with citric acid and cochineal before added to PLA] [62].

To enhance food safety, clove essential oil (CEO) was incorporated into the film matrix. CEO, rich in eugenol, exhibits strong antimicrobial properties against foodborne pathogens, making it an ideal active agent for extending seafood shelf life. Antimicrobial tests demonstrated significant inhibition zones against *Listeria monocytogenes* (16.1 mm) and *Escherichia coli* (12.3 mm), confirming CEO’s effectiveness. CEO’s moisture-sensitive release mechanism was particularly advantageous, as it gradually diffused from the film upon contact with increased humidity, ensuring prolonged antibacterial activity throughout food storage (Figure 6A,B). The in situ packaging application was tested on shrimp, a highly perishable seafood product prone to bacterial spoilage. Fresh shrimp samples were stored in PM20C films at 4 °C for 15 days, and their microbial growth and biochemical changes were monitored. Total viable count (TVC) analysis revealed that shrimp packaged with PM20C maintained TVC levels below the 7 log CFU/mL spoilage threshold for 15 days, compared to only 5 days for conventional plastic packaging. The pH of the shrimp remained below 7.5, while TVC levels increased 10% slower in PM20C films than in unmodified PLA/TPS films, demonstrating superior preservation capabilities [62,63,64].

#### 2.3.3. Industrial Feasibility and Conclusion

The integration of cochineal as a freshness indicator and CEO as an antimicrobial agent resulted in a cost-effective, scalable packaging material with real-time spoilage detection and extended food shelf life. The modified PLA/TPS films exhibited enhanced tensile strength (32.6% improvement), better moisture resistance (11% reduction in water vapor permeability), and increased elasticity, making them suitable for commercial food packaging applications. Unlike many existing intelligent packaging solutions that rely on synthetic pH-sensitive dyes or costly fabrication techniques, the PLA/TPS/CEO/cochineal films provide an industrially viable, biodegradable, and non-toxic alternative. Mohammadi and Fasihi successfully demonstrated that eco-friendly PLA/TPS-based films can function as both active and intelligent packaging materials, addressing food spoilage concerns while reducing plastic waste. Future research should focus on optimizing the release kinetics of essential oils, improving gas barrier properties, and exploring other natural pigments for broader food packaging applications.

### 2.4. Development of PFAS-Free Cellulose Nanofibril-Based Food Packaging for Sustainable Applications

**Figure 7:**
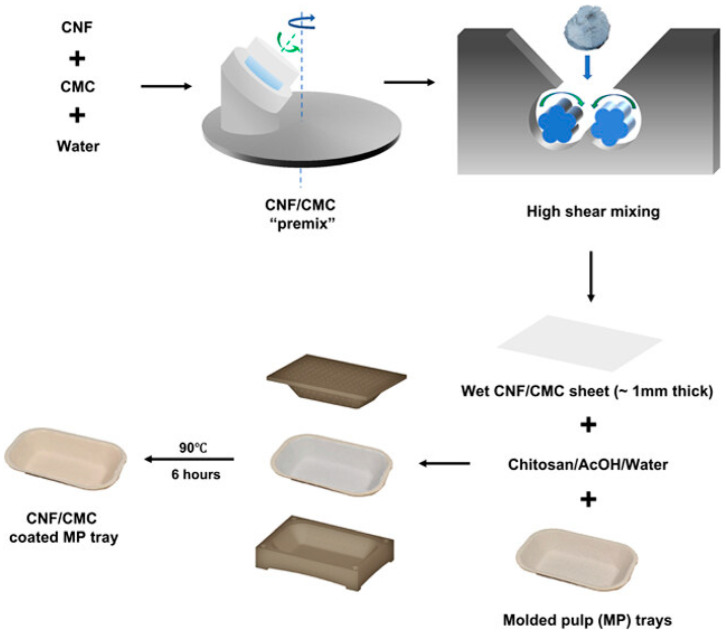
Schematic illustration of CNF/CMC-coated MP tray fabrication process [5].

The increasing environmental concerns surrounding petroleum-based plastic food packaging have driven the search for sustainable alternatives. While paper-based materials offer biodegradability and recyclability, they often lack the necessary barrier properties against air, moisture, and grease, making them unsuitable for long-term food preservation. In response to these challenges, Zhang and Youngblood (2023) [5] developed a cellulose nanofibril (CNF)-coated, PFAS-free, grease-resistant molded pulp (MP) container to serve as an eco-friendly food packaging solution (Figure 7). Their study focused on enhancing the mechanical properties, barrier effectiveness, and sustainability of molded pulp packaging by incorporating a CNF and carboxymethyl cellulose (CMC) paste as a coating material. The novel formulation, applied using an over-molding process, resulted in a uniform and smooth CNF/CMC dry coating approximately 200 µm thick, significantly improving the adhesion, structural integrity, and functional properties of MP food trays.

#### 2.4.1. Structural Characterization and Material Performance

To ensure the viability of the CNF/CMC-coated MP trays for commercial food packaging applications, various analytical techniques were employed. SEM confirmed the uniform dispersion of the CNF/CMC layer, reducing voids and enhancing mechanical stability. FTIR revealed strong hydrogen bonding interactions within the coated structure, confirming its improved interfacial adhesion properties. Additionally, XRD analysis showed that the crystallinity of the coated trays was higher than that of uncoated MP, which contributed to superior moisture resistance and mechanical strength. Chitosan was introduced as an intermediate adhesive layer between the MP substrate and CNF/CMC, further increasing the durability of the coated trays. These modifications not only enhanced the structural cohesion of the material but also made it more adaptable to industrial packaging applications [5,65].

#### 2.4.2. Barrier Properties and Food Preservation

The most significant advantage of the CNF/CMC-coated MP trays lies in their enhanced barrier properties, particularly against grease, moisture, and air. Traditional molded pulp food trays are highly porous, making them vulnerable to oil penetration and moisture absorption. Through standardized TAPPI-T559 grease resistance testing, the researchers found that CNF/CMC-coated samples exhibited exceptional oil repellency, outperforming conventional MP trays that showed immediate staining. Air barrier performance was also significantly improved, as the Gurley air porosity test indicated that the CNF/CMC-coated trays were more than 10,000 times less permeable than uncoated MP trays. Water vapor transmission rate (WVTR) tests further demonstrated that the coated samples maintained excellent resistance under dry conditions, reducing moisture absorption by approximately 90% compared to uncoated MP trays. However, under high-humidity conditions, CNF coatings exhibited reduced effectiveness due to water-induced plasticization, suggesting the need for additional hydrophobic modifications for wet-food applications [66].

**Figure 8:**
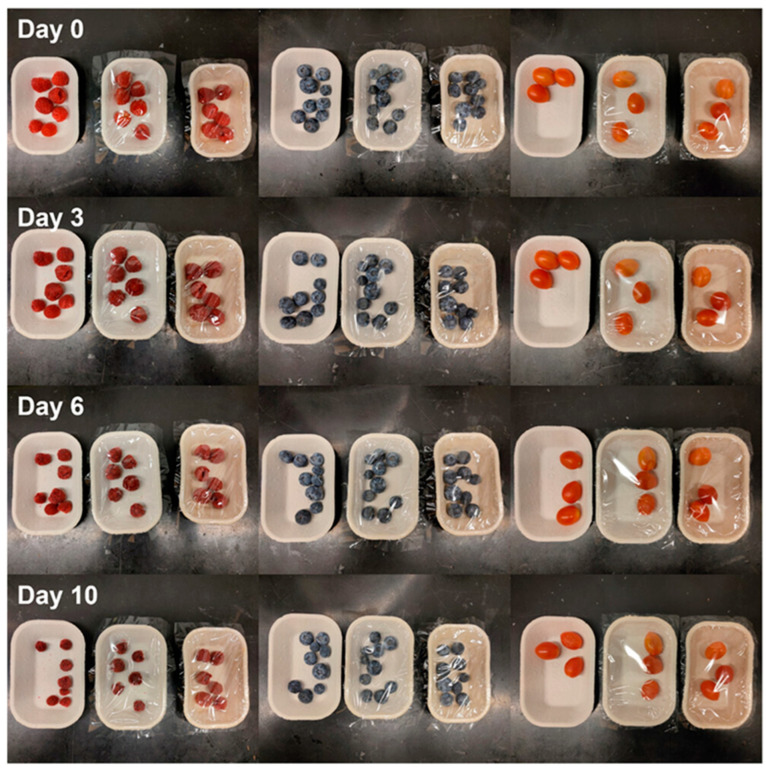
Images of raspberries, blueberries, and cherry tomatoes (left to right) used in shelf-life testing with PLA as the lidding film. Within each set, the samples from left to right are as follows: uncoated without a lid (control), uncoated with a lid, and coated with a lid [5].

The practical application of CNF/CMC-coated trays was evaluated by storing raspberries, blueberries, and cherry tomatoes in the packaging to measure weight loss over time (Figure 8). The results revealed that fruits stored in lidded and coated trays retained significantly more moisture compared to those in uncoated trays, leading to prolonged freshness and extended shelf life. The findings indicated that CNF/CMC-coated MP trays are particularly beneficial for preserving perishable foods, offering a sustainable alternative to traditional plastic food containers.

#### 2.4.3. Industrial Feasibility and Sustainability

Beyond their functional advantages, CNF/CMC-coated MP trays present a viable, scalable, and environmentally friendly packaging alternative. Unlike conventional grease-resistant packaging that relies on per- and polyfluorinated substances (PFASs), which have been linked to serious health and environmental concerns, this innovation provides a biodegradable, non-toxic solution. The coating process is compatible with existing molded fiber production technologies, making it feasible for large-scale industrial adoption without requiring significant alterations to current manufacturing infrastructure. Furthermore, the biodegradable nature of CNF/CMC-coated MP trays supports global efforts to reduce plastic waste and promote compostable packaging solutions [65,66,67].

In conclusion, the study conducted by Zhang and Youngblood highlights the potential of CNF/CMC coatings as a next-generation biodegradable packaging material. By addressing the challenges of grease resistance, moisture barrier effectiveness, and mechanical stability, this research contributes to the development of PFAS-free, environmentally friendly food packaging. Future research should focus on optimizing the hydrophobic properties of CNF coatings, exploring additional natural additives for enhanced functionality, and refining large-scale production techniques to ensure widespread industry adoption. The implementation of such innovations could significantly reduce plastic pollution while ensuring the safety and longevity of food products.

### 2.5. Development of High-Performance Carboxymethyl Cellulose-Based Hydrogel Film for Food Packaging and Preservation

**Figure 9:**
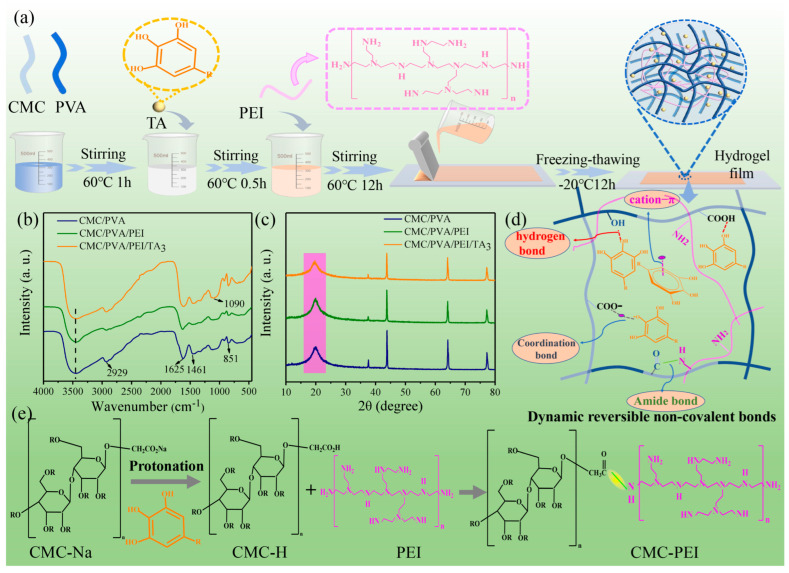
(**a**) Schematic illustration of the fabrication process for CMC-based hydrogel film. (**b**) FTIR and (**c**) XRD spectra of CMC/PVA, CMC/PVA/PEI, and CMC/PVA/PEI/TA. (**d**) Suggested dynamic reversible non-covalent interactions among polymers. (**e**) Amidation reaction between PEI and protonated CMC [68].

The growing demand for sustainable and functional food packaging has led to the exploration of biodegradable alternatives to petroleum-based materials. Traditional food packaging films often suffer from poor mechanical strength, limited stretchability, and inadequate barrier properties, reducing their effectiveness in food preservation. To address these issues, Zhao et al. (2022) [68] developed a highly stretchable and multifunctional sodium carboxymethyl cellulose (CMC)-based hydrogel film incorporating polyvinyl alcohol (PVA), polyethylene imine (PEI), and tannic acid (TA) (Figure 9). This novel hydrogel film exhibits outstanding mechanical properties, strong adhesion, UV-blocking capabilities, self-healing ability, and efficient antioxidant and oxygen barrier functions, making it an ideal candidate for food preservation applications.

#### 2.5.1. Structural and Mechanical Properties

The hydrogel film was engineered with a dynamic three-dimensional (3D) network structure, where reversible non-covalent bonds acted as sacrificial elements to dissipate energy and enhance elasticity. The fabricated CMC/PVA/PEI/TA hydrogel film achieved an elongation of nearly 400%, significantly higher than conventional food packaging films (<50%). FTIR confirmed the presence of amide bonds formed between protonated CMC and PEI in the presence of TA, contributing to the enhanced mechanical properties. SEM analysis revealed a dense and uniform microstructure, minimizing voids and improving overall structural integrity. The hydrogel film demonstrated remarkable resilience under repeated mechanical stress tests, maintaining its flexibility and toughness even after multiple deformation cycles [68].

#### 2.5.2. Barrier Performance and Antioxidant Properties

One of the most critical functions of food packaging is preventing moisture loss and oxidation, both of which contribute to food spoilage. The hydrogel film exhibited strong water vapor and oxygen barrier properties, significantly reducing gas permeability compared to uncoated packaging materials. The addition of tannic acid, a natural antioxidant, further improved the film’s protective capabilities by preventing lipid oxidation, a common cause of food deterioration. The DPPH radical scavenging assay confirmed the high antioxidant potential of the film, with radical scavenging activity increasing proportionally to TA concentration. Additionally, the hydrogel film effectively blocked UV radiation (<400 nm), preventing photodegradation and extending food shelf life.

#### 2.5.3. Self-Healing and Adhesion Performance

The hydrogel film’s dynamic non-covalent bonding network endowed it with excellent self-healing properties, enabling it to recover from mechanical damage without external stimuli. When cut and rejoined, the film seamlessly reformed its structure within one hour, retaining its mechanical integrity. This feature is particularly beneficial for extending packaging durability during handling and transportation. Furthermore, the hydrogel film exhibited strong adhesion to various surfaces, including glass, plastic, metal, and wood, due to the catechol-functionalized tannic acid. The measured adhesive strength reached 234.08 kPa, making it suitable for a wide range of food packaging applications where secure sealing is essential [68,69,70].

#### 2.5.4. Food Preservation Efficacy and Industrial Viability

**Figure 10:**
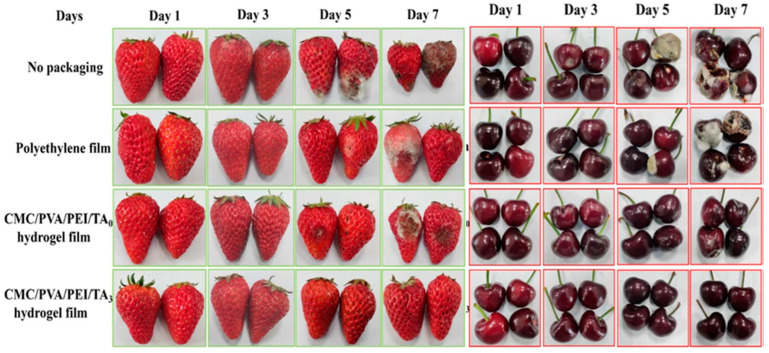
Changes in the visual appearance of strawberries and cherries over the storage period [60].

Packaging fresh strawberries, mangoes, and cherries under ambient conditions tested the practical application of the hydrogel film (Figure 10). The results indicated that fruits stored in the CMC/PVA/PEI/TA_3_ (TA_3_ indicates 0.6 wt.% of tannic acid) hydrogel film maintained their weight, firmness, and color for over a week, outperforming conventional polyethylene packaging and uncoated storage. The film’s ability to regulate moisture retention, suppress microbial growth, and delay oxidative degradation significantly prolonged fruit shelf life, demonstrating its potential as an advanced food preservation system. Moreover, its biodegradability and non-toxic composition ensure environmental sustainability, aligning with the growing global shift toward eco-friendly packaging solutions. Zhao et al. (2022) [68] successfully developed a multifunctional CMC-based hydrogel film that addresses the limitations of conventional food packaging by integrating superior mechanical strength, stretchability, UV-blocking, antioxidant activity, self-healing ability, and strong adhesion. This breakthrough paves the way for next-generation biodegradable food packaging solutions. Future research should focus on optimizing the film’s hydrophobic properties for improved moisture resistance, exploring additional bioactive compounds for antimicrobial functionality, and scaling up the production process for commercial implementation. The adoption of such innovative hydrogel films has the potential to revolutionize food preservation while minimizing plastic waste and environmental impact.

### 2.6. Development of Robust Cellulose/Carboxymethyl Chitosan Composite Films for Fresh Fruit Preservation

**Figure 11:**
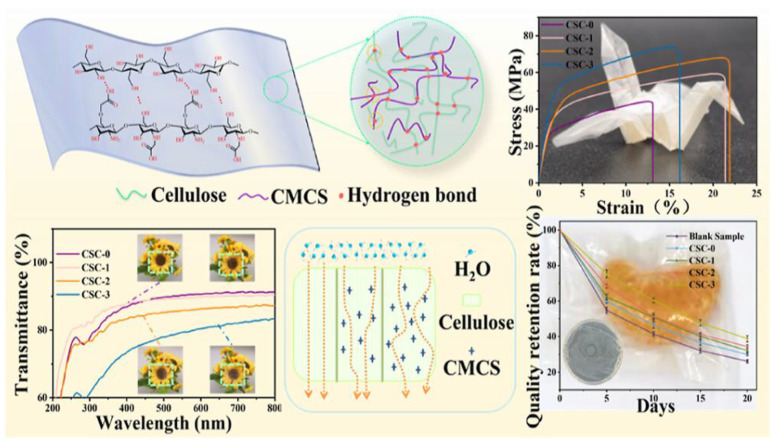
Preparation process of CSCs [33].

The demand for eco-friendly food packaging has grown significantly due to environmental concerns surrounding petroleum-based plastics. Recent advancements in biodegradable materials have introduced cellulose-based composites as promising alternatives. Liao et al. (2023) [33] developed a novel cellulose/carboxymethyl chitosan (CMCS) composite film (CSC) with high transparency, excellent mechanical strength, and antibacterial properties, making it suitable for fresh fruit preservation (Figure 11). The study utilized a simple co-solvent dissolution method to homogeneously blend cellulose with CMCS, forming a robust composite structure. Compared to pure cellulose films, CSC films demonstrated enhanced flexibility, thermal stability, and moisture resistance, effectively prolonging fruit freshness and reducing microbial contamination.

#### 2.6.1. Structural and Mechanical Properties

The fabrication of CSC films involved the dissolution of cellulose and CMCS in a zinc chloride aqueous system, followed by solution casting and regeneration processes. This method resulted in a homogeneous molecular dispersion of CMCS within the cellulose matrix, improving mechanical properties through increased hydrogen bonding interactions. FTIR confirmed strong physical cross-linking between CMCS and cellulose, while SEM revealed a dense, uniform surface morphology, minimizing structural voids.

The mechanical performance of CSC films was significantly improved by CMCS incorporation. Compared to pure cellulose films (CSC-0), the tensile strength and elongation at the break of CSC-3 increased by 68.8% and 23.2%, respectively. The enhanced flexibility and toughness make these films highly suitable for food packaging applications, where durability is crucial for handling and transportation. Moreover, thermal stability tests demonstrated that the onset degradation temperature of CSC-3 was 10.3 °C higher than CSC-0, confirming the improved resistance to heat-induced decomposition [33].

#### 2.6.2. Barrier and Antibacterial Performance

One of the key challenges in food packaging is maintaining moisture and oxygen barrier properties to extend shelf life. CSC films exhibited excellent water vapor and oxygen resistance due to their dense hydrogen bonding network. Water vapor permeability (WVP) and water absorption tests showed that CSC-3 had 39.1% lower WVP and 54.6% reduced water uptake compared to unmodified cellulose films. This enhanced barrier performance effectively reduces fruit dehydration and nutrient loss, making CSC films ideal for fresh produce storage.

In addition to their physical barrier properties, CSC films demonstrated strong antibacterial activity against *Staphylococcus aureus* and *Escherichia coli*, two common foodborne pathogens. The presence of CMCS, which contains positively charged amino groups, enabled electrostatic interactions with bacterial cell membranes, leading to membrane disruption and bacterial cell death. The inhibition zone tests revealed that CSC films effectively suppressed microbial growth, thereby enhancing food safety and extending the shelf life of packaged fruits [71,72].

#### 2.6.3. Food Preservation and Industrial Feasibility

**Figure 12:**
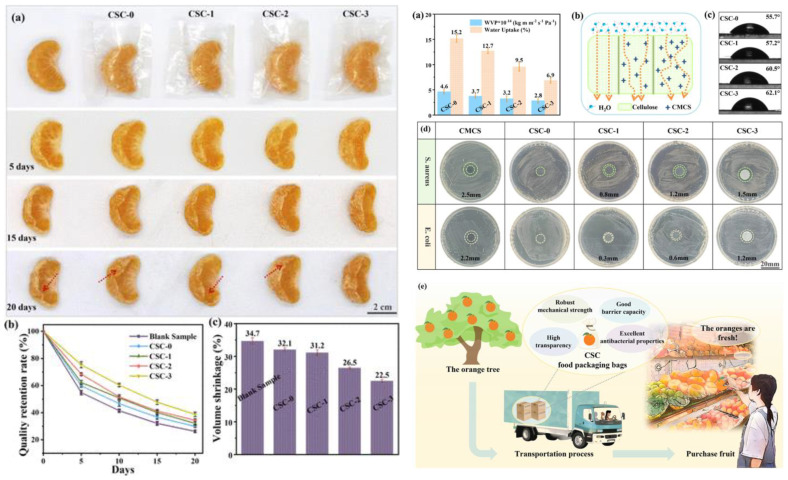
(**A**) (a) Comparison of samples used for packing oranges. (b) Retention of quality in oranges. (c) Shrinkage in volume of oranges. (**B**) (a) Water absorption and barrier properties against water/water vapor. (b) Potential water molecule penetration routes. (c) Water contact angle measurements for CSC-0, CSC-1, CSC-2, and CSC-3 films. (d) Antibacterial activity against *S. aureus* and *E. coli*. (e) Simulation results for CSCs in fruit packaging [33].

To evaluate the effectiveness of CSC films in real-world applications, fresh oranges were wrapped in the composite films and stored at room temperature. Over a 20-day period, CSC-packaged oranges exhibited significantly lower weight loss and volume shrinkage compared to unwrapped samples (Figure 12A). The quality retention rate of CSC-3-wrapped oranges remained 61.2%, whereas unwrapped oranges lost up to 70.8% of their initial weight. These findings confirm that CSC films effectively minimize moisture loss, reduce oxidation, and maintain fruit freshness over extended storage durations.

The industrial feasibility of CSC films is supported by their simple and scalable fabrication process. Unlike complex cross-linking methods that require multiple chemical treatments, the co-solvent dissolution technique used in this study provides a cost-effective and environmentally friendly alternative. Additionally, CSC films are fully biodegradable and compostable, aligning with the global push for sustainable packaging solutions (Figure 12B). Liao et al. (2023) [33] successfully developed a high-performance cellulose/CMCS composite film that addresses key limitations in biodegradable food packaging. With superior mechanical strength, moisture resistance, and antibacterial properties, CSC films offer a viable replacement for traditional plastic packaging in fresh fruit preservation. Future research should focus on optimizing the hydrophobic properties of CSC films to further improve moisture resistance, incorporating natural antioxidants to enhance oxidative stability, and scaling up production for commercial applications. These advancements will further position CSC films as a sustainable, effective, and commercially viable food packaging solution, contributing to reduced plastic waste and improved food safety.

#### 2.6.4. Emerging Chitosan-Based Films for Food Packaging Applications

In response to increasing environmental concerns surrounding petroleum-based plastics, chitosan-based films have gained significant attention for their potential in sustainable food packaging. Wang et al. (2025) [73] comprehensively reviewed recent advancements in chitosan-based films, highlighting their fabrication methods, functional enhancements, and applications in food preservation. Chitosan, a derivative of chitin, offers biodegradability, antimicrobial properties, and excellent film-forming ability, making it a promising alternative for food packaging materials. Researchers have focused on improving its physical and functional properties by incorporating nanomaterials, biopolymers, and natural extracts to enhance its mechanical strength, water resistance, and active packaging capabilities.

#### 2.6.5. Fabrication and Functional Enhancement of Chitosan Films

**Figure 13:**
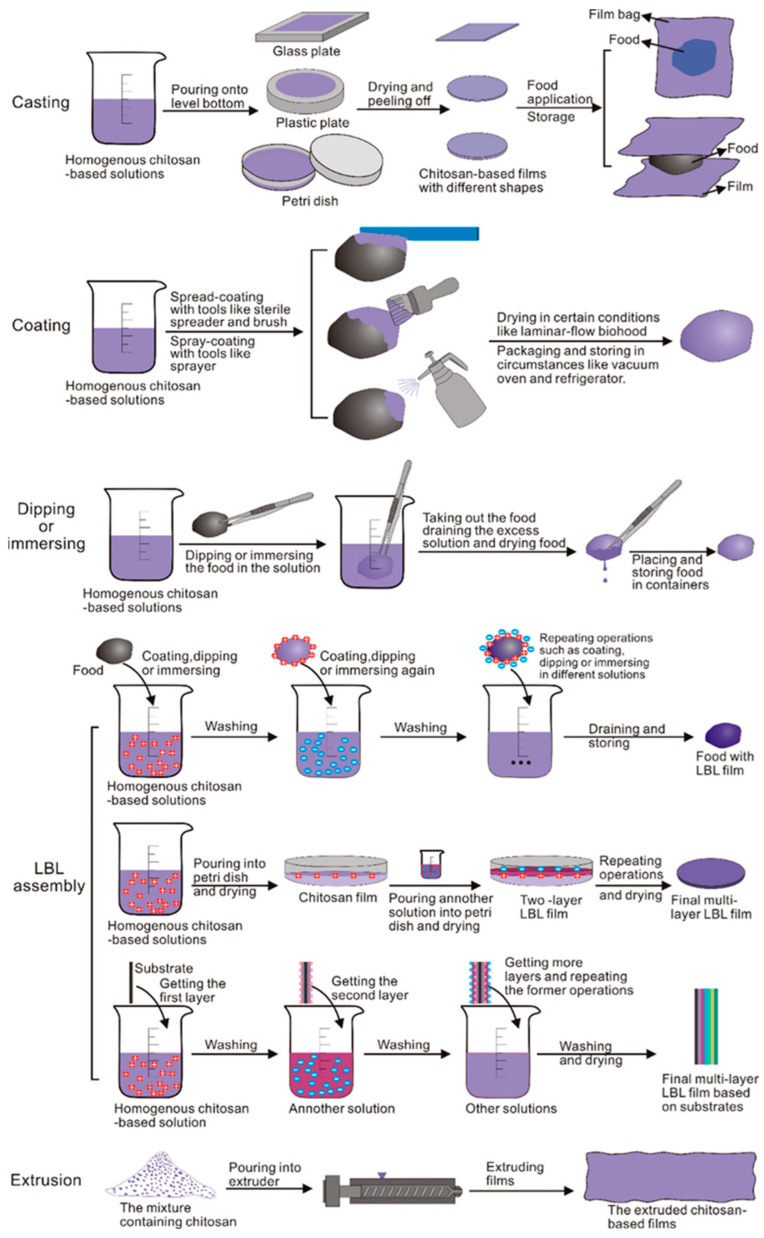
Different methods to fabricate the chitosan-based films [73].

Chitosan-based films can be fabricated using various methods, including casting, coating, layer-by-layer assembly, dipping, and extrusion. These techniques influence the film’s structure, flexibility, and overall performance (Figure 13). Pure chitosan films are widely used due to their natural antimicrobial and antioxidant activities, but their brittleness and moisture sensitivity have led to the development of composite films. Researchers have combined chitosan with biopolymers (starch, cellulose, alginate), proteins (gelatin, casein), and synthetic polymers (polyvinyl alcohol, polylactic acid) to improve its mechanical and barrier properties. Additionally, plasticizers like glycerol and sorbitol enhance chitosan’s flexibility and film-forming capabilities [74].

#### 2.6.6. Applications in Active and Intelligent Packaging

**Figure 14:**
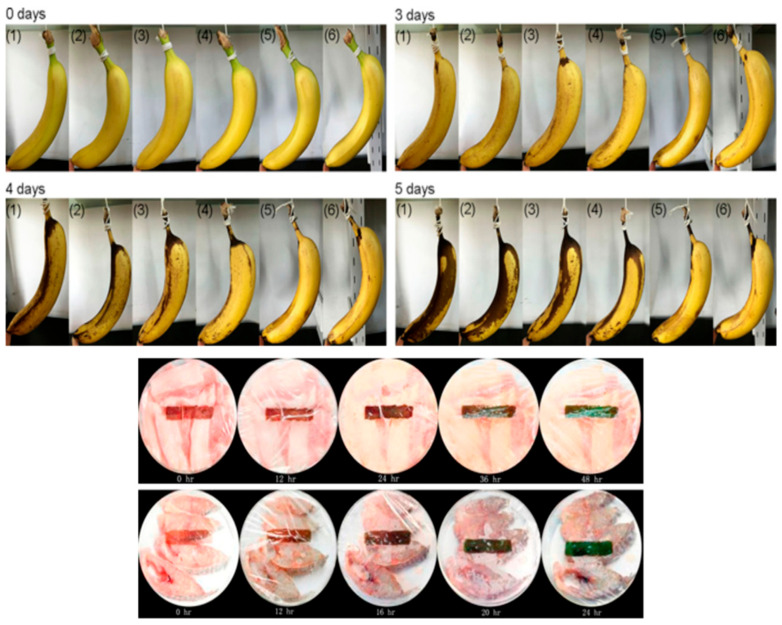
Comparison of uncoated bananas (1) and bananas coated with CMC100 film (2), HTCC40/CMC60 film (3), HTCC70/CMC30 film (4), HTCC90/CMC10 film (5), and HTCC100 film (6). CMC refers to carboxymethyl cellulose, and HTCC refers to 2-N-hydroxypropyl-3-trimethylammonium chloride chitosan. The application of the sensing film as a freshness indicator for pork and fish [73].

The versatility of chitosan-based films allows them to function beyond passive packaging, incorporating active components that improve food safety and shelf life. Antimicrobial chitosan films, often enhanced with essential oils, silver nanoparticles, or natural extracts, have shown significant efficacy against foodborne pathogens, preventing microbial spoilage in perishable foods like meat, fish, and dairy products. Barrier films with nanocellulose or montmorillonite clay have improved moisture resistance, extending the shelf life of packaged foods by reducing oxygen and water vapor permeability. Additionally, sensing films embedded with pH-sensitive dyes or nanoparticles have emerged as intelligent packaging solutions that can indicate food freshness through visible color changes in response to spoilage-related pH shifts (Figure 14). The integration of chitosan with functional additives has led to the development of multifunctional food packaging films capable of preserving food quality while reducing environmental impact. Despite these advancements, challenges remain, including cost-effectiveness, large-scale production feasibility, and maintaining film stability in varying storage conditions. Future research should focus on optimizing biodegradability, recyclability, and large-scale industrial applications, ensuring that chitosan-based films can replace conventional plastic packaging while meeting the growing demand for sustainable and intelligent food packaging solutions [73,74,75].

### 2.7. Triboelectric Nanogenerators for Intelligent Food Packaging and Energy Harvesting

**Figure 15:**
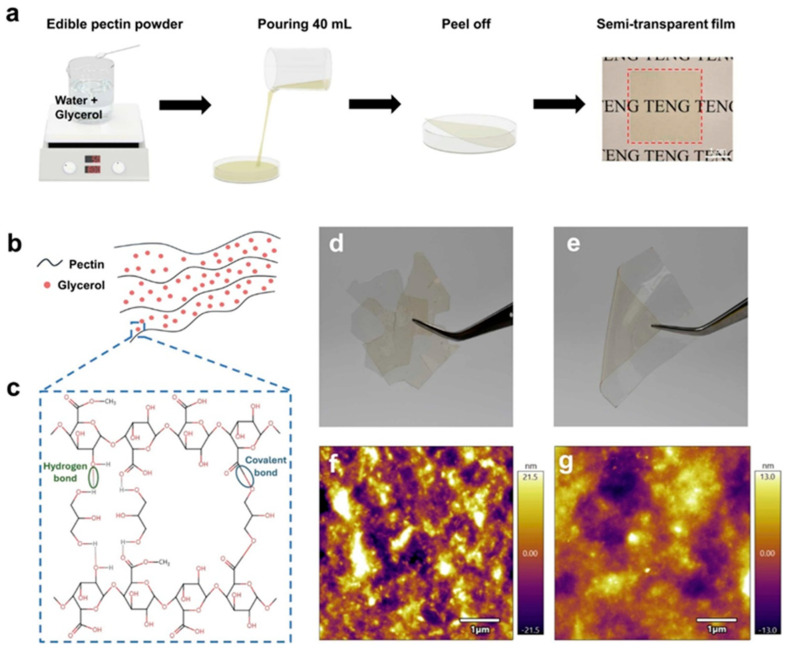
(**a**) Fabrication process of pectin–glycerol (PG) films. (**b**) Schematic and (**c**) mechanism illustrations showing glycerol’s plasticizing effect in PG films. Digital images of (**d**) PG film without glycerol (PG-0) and (**e**) PG film with 50% glycerol relative to pectin content (PG-50). (**f**,**g**) Atomic force microscopy images of the surface of (**f**) PG-0 and (**g**) PG-50 films [76].

In recent years, the need for sustainable and intelligent food packaging has grown due to increasing concerns over food waste and environmental sustainability. Jin et al. (2025) [76] introduced an innovative approach by developing triboelectric nanogenerator (TENG)-based food packaging that can both harvest mechanical energy and monitor food quality in real time. By integrating carbohydrate polymer-based triboelectric layers with glycerol as a plasticizer, the researchers created a multifunctional system capable of detecting humidity-induced quality changes in low-moisture foods (Figure 15). This approach presents a novel solution to the limitations of conventional food packaging, offering dynamic freshness monitoring without the need for external power sources.

#### 2.7.1. Fabrication and Properties of Pectin-Based TENGs

The study utilized pectin, a carbohydrate polymer derived from citrus peel waste, as the core material for the triboelectric layers. To enhance the flexibility and triboelectric performance of the films, varying concentrations of glycerol were incorporated as a plasticizer. The films were fabricated using a solution-casting method, and their physical and chemical properties were extensively characterized. FTIR confirmed strong hydrogen bonding between pectin and glycerol, while atomic force microscopy (AFM) revealed that higher glycerol content reduced surface roughness, leading to improved film flexibility. The optimal composition, containing 70% glycerol relative to pectin, exhibited the highest triboelectric output, making it ideal for energy-harvesting applications.

#### 2.7.2. Energy Harvesting and Humidity-Sensitive Food Monitoring

**Figure 16:**
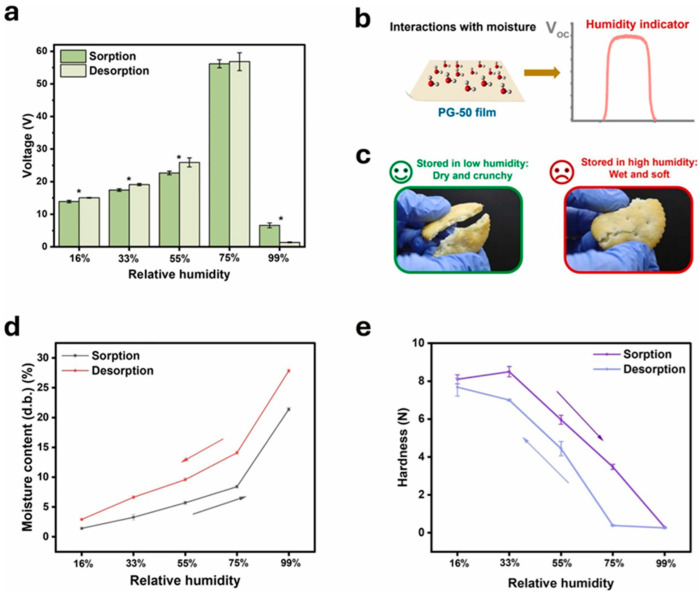
(**a**) Voltage output of pectin–glycerol film-based triboelectric nanogenerators (P-TENGs), where one triboelectric layer is PG-50 (pectin–glycerol film with 50% glycerol by weight), after water absorption and desorption at varying relative humidity (RH) levels. Asterisks denote significant differences in voltage outputs between the water-sorption and water-desorption processes at specific RH values (*p* < 0.05). (**b**) Operating principle of a P-TENG used as a humidity sensor. (**c**) Changes in cracker quality when stored at low vs. high RH. (**d**) Dry-basis moisture content of crackers stored at different RH levels. Black: water sorption; red: water desorption. (**e**) Hardness variation in crackers stored at different RH levels. Purple: water sorption; blue: water desorption [76].

One of the most significant findings of this research was the ability of pectin-based TENGs to convert mechanical energy into electricity. When subjected to external mechanical force, the TENGs generated a peak voltage of 55 V and a power density of 8.75 mW/m^2^ (Figure 16a–e). These devices successfully powered small electronics, such as LED lights, calculators, and hygrometers, demonstrating their potential for self-powered food packaging applications. Beyond energy harvesting, the triboelectric response of the films was highly sensitive to humidity changes. As pectin is naturally hygroscopic, the films absorbed moisture from the environment, altering their triboelectric performance. This property was leveraged to monitor the quality of crackers, a representative low-moisture food, by detecting changes in moisture content and texture [77].

#### 2.7.3. Triboelectric Food-Quality Sensors for Intelligent Packaging

To make this technology practical for food packaging, the researchers designed a triboelectric food-quality sensor (TFQS) integrated into cracker packaging. The sensor consisted of a pectin-based TENG paired with a graphical user interface (GUI) that displayed real-time food quality based on voltage output. The system categorized food freshness into three levels: good quality (10–20 V), degrading quality (21–30 V), and spoiled (above 50 V or below 10 V). This approach addresses the shortcomings of traditional expiration date labels, which fail to account for storage conditions and often lead to unnecessary food waste. By providing real-time freshness feedback, this technology empowers consumers to make informed decisions about food consumption, thereby extending shelf life and reducing waste. The integration of biodegradable triboelectric materials into food packaging marks a significant advancement in intelligent packaging technologies. This study demonstrated that carbohydrate polymer-based TENGs offer a cost-effective, scalable, and environmentally friendly alternative to conventional freshness indicators [76,77,78]. Unlike synthetic colorimetric sensors or electronic monitors requiring external power, these self-powered devices provide dynamic monitoring while remaining sustainable. Future research should explore optimizing gas barrier properties, refining the release kinetics of moisture-sensitive triboelectric layers, and expanding applications to different food types. By enhancing real-time food quality monitoring, this technology has the potential to revolutionize food packaging, promoting both sustainability and consumer safety.

### 2.8. Development of Active Antibacterial CEO/CS@PLA Nonwovens for Food Preservation

**Figure 17:**
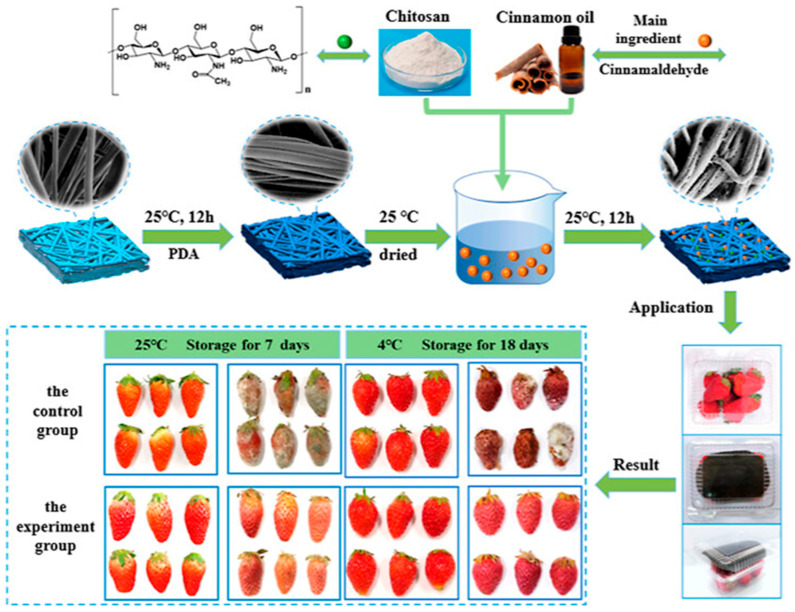
Preparation and fabrication route of CEO/CS@PLA MB [79].

With increasing concerns over food safety and environmental sustainability, biodegradable antibacterial materials have gained significant attention in food packaging applications. Fresh foods, particularly small-sized fruits like strawberries, are highly perishable due to their high metabolic activity and vulnerability to microbial contamination. Traditional food packaging materials often fail to provide adequate antibacterial protection, leading to significant food waste and health risks. To address these challenges, Sun et al. (2023) [79] developed a biodegradable melt-blown nonwoven (MB) based on polylactic acid (PLA), chitosan (CS), and cinnamon essential oil (CEO). This innovative CEO/CS@PLA MB material combines the benefits of PLA’s biodegradability, CS’s antimicrobial properties, and CEO’s antioxidant effects (Figure 17), offering an effective solution for food preservation while reducing environmental impact.

#### 2.8.1. Fabrication and Structural Properties

The CEO/CS@PLA nonwovens were fabricated using a polydopamine (PDA)-assisted surface modification technique, followed by the deposition of CS and CEO through a one-pot method. PDA was employed to enhance the surface reactivity of PLA, improving the adhesion of CS and CEO. SEM analysis revealed that CEO/CS@PLA MB exhibited a rougher surface and larger fiber diameter than unmodified PLA MB, contributing to improved antibacterial performance. However, the incorporation of CEO led to a reduction in air permeability and a decrease in tensile strength while significantly increasing the elongation at break, making the material more flexible. Fourier-transform infrared spectroscopy (FTIR) confirmed the presence of CEO and CS on the PLA MB surface, while X-ray diffraction (XRD) analysis indicated a decrease in crystallinity with increasing CEO content.

#### 2.8.2. Antibacterial Performance and Food Preservation Efficiency

**Figure 18:**
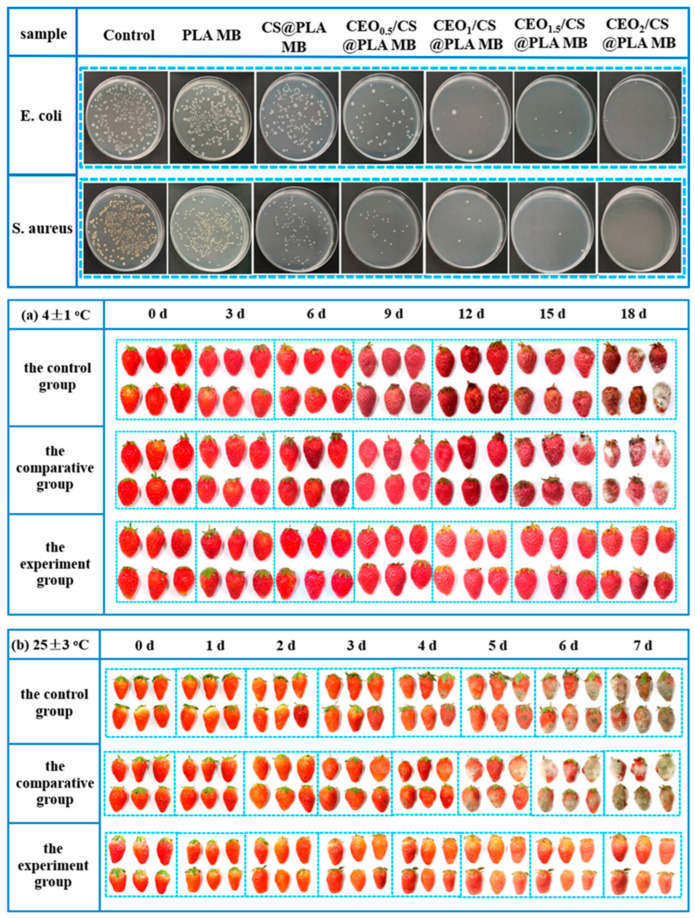
Variation in colony count of *E. coli* and *S. aureus* on PLA MB, CS@PLA MB, and CEO/CS@PLA MB with different CEO concentrations. Visual appearance of strawberries from three groups stored at (**a**) 4 ± 1 °C and (**b**) 25 ± 3 °C [79].

The antibacterial effectiveness of CEO/CS@PLA MB was evaluated against Escherichia coli and Staphylococcus aureus, two common foodborne pathogens. Results showed a synergistic effect between CS and CEO, leading to 99.98% and 99.99% inhibition of *E. coli* and *S. aureus*, respectively, when the CS-to-CEO ratio was 1:2. This significant antibacterial efficiency was attributed to the cationic nature of CS, which disrupts bacterial membranes, and cinnamaldehyde (CIN), the active compound in CEO, which interferes with bacterial metabolism. To assess its real-world applicability, CEO/CS@PLA MB was tested for the preservation of fresh strawberries. The nonwoven films effectively inhibited microbial growth, reducing decay and extending shelf life by preventing weight loss, maintaining fruit firmness, and slowing oxidative deterioration. Strawberries stored with CEO/CS@PLA MB exhibited (Figure 18) significantly lower decay rates and higher antioxidant retention compared to those stored in conventional packaging.

The successful development of CEO/CS@PLA MB demonstrates the potential of biodegradable, antibacterial nonwovens in active food packaging. Unlike conventional plastic-based packaging, these nonwovens offer a cost-effective, scalable, and environmentally friendly alternative. The combination of PLA’s biodegradability, CS’s antimicrobial properties, and CEO’s natural preservation effects makes this material a promising solution for extending the shelf life of perishable foods. Future research should focus on optimizing the controlled release of CEO, enhancing mechanical durability, and expanding applications to other food products such as dairy and meat. By integrating natural antibacterial agents into biodegradable packaging, CEO/CS@PLA MB contributes to a more sustainable and effective food preservation strategy, addressing both food safety concerns and plastic pollution [79,80,81].

### 2.9. Biodegradable and Flexible Nanoporous Films for Active Food Packaging

Nanotechnology has significantly advanced the development of active food packaging systems, enabling functionalities beyond traditional packaging materials. One of the primary goals of modern food packaging is to extend shelf life while maintaining safety and sustainability. Conventional materials such as polyethylene terephthalate (PET) often lack moisture control and antimicrobial properties, leading to rapid spoilage and food waste. In response to these challenges, Kim et al. (2022) [82] developed biodegradable and flexible nanoporous polycaprolactone-based (FNP) films using a plasma-enabled nanofabrication strategy. These films aim to improve food storage quality by regulating moisture levels, preventing microbial contamination, and providing a sustainable alternative to plastic packaging.

#### 2.9.1. Fabrication and Structural Characterization

**Figure 19:**
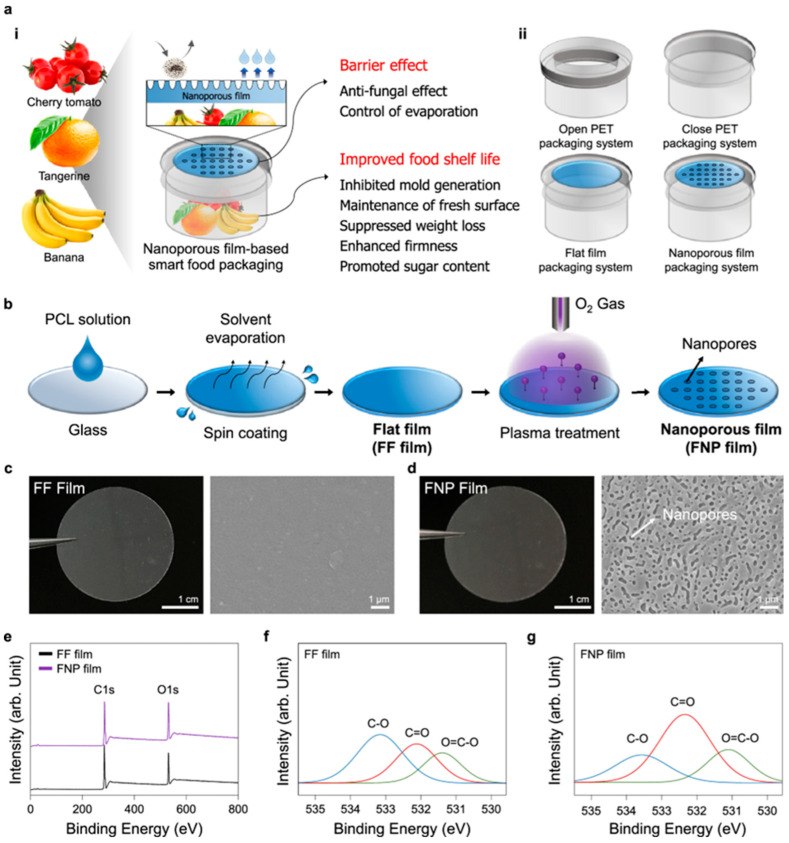
Schematic of flexible nanoporous polycaprolactone-based (FNP) films for active food packaging: (**a**) (i) Overview of study approaches and strategies; (ii) design of packaging systems, including open and closed PET (control), flexible flat PCL (FF), and FNP-based packaging for preserving cherry tomatoes, tangerines, and bananas. (**b**) FNP film fabrication via O_2_ plasma modification of FF films. (**c**,**d**) FE-SEM images of FF (**c**) and FNP (**d**) film surfaces. (**e**) XPS scans of FF and FNP films. (**f**,**g**) High-resolution O1s XPS spectra of FF (**f**) and FNP (**g**) films [82].

The FNP films were produced by modifying flexible flat polycaprolactone (FF) films using oxygen plasma treatment. This process introduced nanoporous structures ranging from 50 to 400 nm, significantly enhancing surface roughness and gas permeability. Fourier-transform infrared spectroscopy (FTIR) and X-ray photoelectron spectroscopy (XPS) confirmed successful chemical modifications induced by plasma treatment, while scanning electron microscopy (SEM) revealed improved porosity compared to untreated films (Figure 19). The permeability characteristics of these films were also examined, with FNP films demonstrating a higher oxygen transmission rate (5382 cm^3^/m^2^/day) and water vapor transmission rate (750 g/m^2^/day) compared to traditional PET packaging, which had significantly lower permeability values. These modifications allowed for better moisture regulation, preventing excessive humidity buildup that often leads to mold growth in packaged food products [83].

#### 2.9.2. Food Preservation and Antimicrobial Performance

The FNP films were evaluated for their ability to preserve different types of perishable foods, including cherry tomatoes, tangerines, and bananas. In storage trials, cherry tomatoes wrapped in FNP films exhibited zero mold growth for 13 days, while those in closed PET containers developed mold within three days due to trapped moisture. Freshness indicators such as firmness and weight retention showed superior results in FNP-packaged tomatoes, with a higher firmness of 24.7 N and lower weight loss (2.79%) compared to open PET packaging, where weight loss reached 8.32%. Similar trends were observed in tangerines, where FNP films prevented mold formation for 17 days, unlike closed PET packaging, which led to complete fruit decay. The weight retention in FNP-packaged tangerines was significantly better (2.45%) than in open PET (13.49%), confirming the film’s ability to balance moisture content and prevent dehydration. For bananas, the FNP films effectively delayed ripening by reducing black spots and preventing rapid starch hydrolysis, leading to slower sugar conversion and extended shelf life. The total soluble solid (TSS) values in FNP-packaged bananas remained lower, indicating delayed ripening compared to conventional packaging [84].

**Figure 20:**
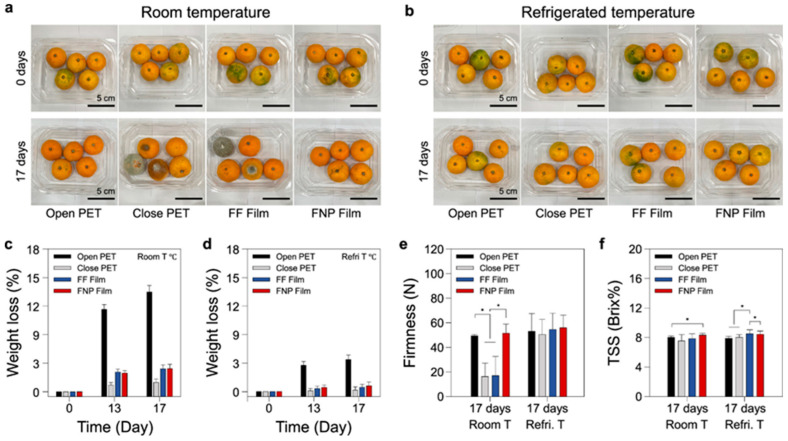
Impact of flexible nanoporous polycaprolactone (FNP) film on the storage quality of tangerines: (**a**,**b**) Mold growth on tangerines stored at 25 °C (**a**) and 4 °C (**b**) for 17 days. (**c**,**d**) Tangerine weight loss at 25 °C (**c**) and 4 °C (**d**) over 17 days. (**e**) Firmness of tangerines stored at 25 °C for 17 days. (**f**) Total soluble solid (TSS) content (°Brix) of tangerines stored at 25 °C for 17 days [82]. * indicate statistical significance.

The incorporation of nanoporous structures into biodegradable PCL films offers a scalable, cost-effective solution for food packaging industries seeking sustainable alternatives. Unlike synthetic coatings or multilayer packaging solutions that are expensive and difficult to recycle, FNP films provide an eco-friendly approach with enhanced barrier properties and active food preservation features. The study demonstrates that these films can be seamlessly integrated into existing packaging systems while offering superior performance in maintaining freshness and reducing spoilage (Figure 20). Future research should focus on optimizing gas barrier properties, refining moisture control mechanisms, and exploring additional natural additives for improved antimicrobial efficacy. Expanding the application of these films to other food categories, such as dairy and meat products, could further solidify their role in next-generation sustainable food packaging solutions [82,83,84].

### 2.10. Packaging Materials Based on Bacterial Nanocellulose

Bacterial nanocellulose (BNC) has emerged as a promising material for sustainable food packaging due to its unique structural and functional properties. Produced by bacteria such as Gluconacetobacter xylinus, BNC features a highly pure, three-dimensional nanofibrillar structure that offers excellent mechanical strength, high crystallinity, water retention capacity, and biocompatibility. Its biodegradability and edibility make it an environmentally friendly alternative to conventional synthetic polymers used in packaging. Various studies have explored the integration of antimicrobial agents into BNC to enhance its functionality. For instance, in the study conducted by Padrão et al. (2016) [85], BNC films were functionalized with bovine lactoferrin (bLF), a natural antimicrobial protein. These modified films (BC + bLF) showed significant bactericidal activity against Escherichia coli and Staphylococcus aureus, making them suitable for direct contact with perishable foods like fresh sausages. The films demonstrated favorable water vapor permeability, mechanical properties, and no cytotoxicity when tested on fibroblasts, further supporting their use as edible antimicrobial packaging.

Another innovative approach in BNC-based packaging involves the formation of nanocomposites using chitosan and nanoparticles. In the work conducted by Salari et al. (2018) [86], bacterial cellulose nanocrystals (BCNCs) were obtained via acid hydrolysis and incorporated into chitosan films alongside silver nanoparticles (AgNPs). The addition of BCNCs and AgNPs significantly improved the films’ mechanical, thermal, and barrier properties. FTIR and XRD analyses confirmed strong interactions between chitosan and BCNCs and the crystalline nature of the composite films. Moreover, the films exhibited powerful antimicrobial activity against a broad range of foodborne pathogens. These enhancements suggest that BCNC/AgNP-reinforced chitosan films can serve as active packaging materials, offering improved preservation and safety for packaged foods. The inclusion of nanofillers also provided a more controlled water vapor transmission rate and reduced film solubility, further supporting their application in food packaging systems where moisture control is critical.

Stroescu et al. (2018) [87] presented another functional application of BNC in the development of antimicrobial food pads. These pads were formulated using a composite of bacterial cellulose, xanthan gum, and carboxymethylcellulose to form superabsorbent hydrogels. Designed to absorb exudates from fresh meat, poultry, or produce, these hydrogels help reduce microbial contamination and food spoilage. The antimicrobial functionality was enhanced with the inclusion of natural agents like thyme essential oil and potassium sorbate. Tested against various microbial strains including *E. coli* and *Candida utilis*, the pads demonstrated effective antimicrobial action. Importantly, the materials used are fully biodegradable and align with the increasing consumer and regulatory demand for natural, safe, and eco-friendly packaging solutions. The study emphasized that such cellulose-based hydrogels not only extend shelf life and improve food hygiene but also address the growing concerns regarding synthetic additives and plastic waste.

## 3. Conclusions and Perspective

The advancement of functional biopolymer films with antimicrobial and antioxidant properties presents a transformative step toward sustainable, active food packaging. In this review, we explored several classes of bio-based films, including those derived from cellulose, chitosan, polylactic acid (PLA), and composite formulations incorporating essential oils, polyphenols, and nanoparticles. Each of these technologies offers unique advantages yet also presents distinct challenges that need to be addressed to achieve broader industrial adoption. Cellulose-based films stand out for their abundance, biodegradability, and excellent film-forming capabilities. However, their inherent hydrophilicity and limited barrier properties require enhancement, often achieved through nanomaterial integration or surface modification. In contrast, chitosan-based films offer intrinsic antimicrobial activity and good oxygen barrier performance, but they suffer from poor mechanical strength and water sensitivity, which restrict their independent application in high-moisture food environments. PLA-based films are recognized for their compostability and favorable mechanical properties. Yet, their relatively high production cost and brittleness, along with limited intrinsic antimicrobial functions, necessitate the addition of bioactive compounds. Blending PLA with other polymers or reinforcing it with natural extracts and nanoparticles can improve its performance, but this may affect its biodegradability and food safety compliance. The use of plant-derived polyphenols and essential oils as active agents contributes both antioxidant and antimicrobial benefits. However, challenges such as volatility, strong aroma, and potential interactions with food matrices require the development of controlled release mechanisms, such as nanoencapsulation or multilayer structures. Nanoparticles, including silver, zinc oxide, and nanoclays, significantly enhance the mechanical and barrier properties of biopolymer films, yet raise concerns about migration, toxicity, and regulatory acceptance. Intelligent packaging systems, such as pH-responsive films and freshness indicators, offer real-time monitoring of food quality, bridging the gap between passive packaging and active consumer engagement. While promising, these systems are often in the experimental stage and need further validation for reliability, cost-effectiveness, and consumer usability in commercial settings. From a production standpoint, emerging fabrication methods such as solution blow spinning, melt blending, and plasma-assisted nanofabrication offer scalable and cost-effective routes to produce advanced functional films. Yet, industrial implementation demands optimization to ensure consistency, reproducibility, and compatibility with existing packaging lines.

Regulatory approval and safety assessment remain critical barriers for active and intelligent packaging. The migration of active agents must be thoroughly evaluated under various storage conditions. Although natural bioactive compounds are generally perceived as safer alternatives, their stability, interaction with food components, and long-term effects require deeper investigation and standardized testing protocols. In the broader context of sustainability, the integration of these biopolymers into circular economy models necessitates a clear understanding of their environmental impact post-use. While materials like cellulose, chitosan, and PLA are biodegradable, their decomposition behavior under real-world composting or landfill conditions must be validated. Consumer acceptance and industry readiness also play pivotal roles. Public education, policy incentives, and industry–academic collaborations are crucial in accelerating the shift from conventional petroleum-based plastics to bio-based, functional alternatives. In conclusion, each functional biopolymer system discussed in this review brings specific benefits to the table—ranging from biodegradability and mechanical robustness to antimicrobial efficacy and smart sensing. However, no single system is without limitations. A multidisciplinary approach that combines the strengths of various materials and technologies, alongside focused innovation in safety, manufacturing, and sustainability, is essential to unlock the full potential of these solutions. With continued research and cross-sector collaboration, functional biopolymer films are well positioned to redefine the future of food packaging—reducing plastic waste, extending shelf life, and safeguarding public health.

## Figures and Tables

**Table 1:** Advantages of improved food packaging.

Advantage	Description	Comparison with Other Methods
Barrier Function [22]	Blocks moisture, oxygen, light, and contaminants.	Thermal or pressure processing can kill microbes, but they do not protect post-treatment.
Shelf Life Extension [23]	Modified Atmosphere Packaging (MAP) slows microbial growth and oxidation.	Unlike natural preservatives, packaging can precisely control gas ratios over time.
Reduced Need for Additives [24]	Minimizes use of chemical preservatives, appealing to “clean label” trends.	Consumers prefer fewer artificial ingredients—packaging helps achieve that.
Temperature Insulation [25]	Some packaging can help maintain thermal conditions (e.g., thermal liners).	Cold chain systems need external power; packaging enhances passive protection.
Smart Monitoring Capabilities [25]	Embedded indicators can detect gas changes, microbial growth, or temp deviations.	Irradiation or preservatives do not provide real-time feedback.
Improved Logistics and Handling [26]	Better stacking, cushioning, and protection during transport.	Other methods do not address mechanical spoilage or crushing.
Sustainability and Waste Reduction [27]	Recyclable/compostable packaging and longer shelf life help reduce food waste.	Irradiation or HPP requires energy; biodegradable packaging offers a low-impact option.
Consumer Convenience [28]	Easy-open, resealable, portion-controlled, or microwavable packaging enhances usability.	Thermal or biocontrol methods do not offer functional consumer benefits.
Cost-Efficiency at Scale [29]	Once implemented, active packaging can be cheaper than continual application of preservatives.	High-pressure or irradiation methods can be expensive for small-scale producers.
